# Enhancing magnetic resonance imaging-driven Alzheimer’s disease classification performance using generative adversarial learning

**DOI:** 10.1186/s13195-021-00797-5

**Published:** 2021-03-14

**Authors:** Xiao Zhou, Shangran Qiu, Prajakta S. Joshi, Chonghua Xue, Ronald J. Killiany, Asim Z. Mian, Sang P. Chin, Rhoda Au, Vijaya B. Kolachalama

**Affiliations:** 1 Section of Computational Biomedicine, Department of Medicine, Boston University School of Medicine, 72 E. Concord Street, Evans 636, Boston, MA 02118 USA; 2 Department of Computer Science, College of Arts & Sciences, Boston University, Boston, MA USA; 3 Department of Physics, College of Arts & Sciences, Boston University, Boston, MA USA; 4 Department of Anatomy and Neurobiology, Boston University School of Medicine, Boston, MA USA; 5 Department of General Dentistry, Boston University School of Dental Medicine, Boston, MA USA; 6 Department of Radiology, Boston University School of Medicine, Boston, MA USA; 7 Department of Neurology, Boston University School of Medicine, Boston, MA USA; 8 Boston University Alzheimer’s Disease Center, Boston, MA USA; 9 Department of Brain and Cognitive Science, Massachusetts Institute of Technology, Cambridge, MA USA; 10 Center of Mathematical Sciences & Applications, Harvard University, Cambridge, MA USA; 11 The Framingham Heart Study, Boston University School of Medicine, Boston, MA USA; 12 Department of Epidemiology, Boston University School of Public Health, Boston, MA USA; 13 Faculty of Computing & Data Sciences, Boston University, Boston, MA USA

**Keywords:** Alzheimer’s disease, Magnetic resonance imaging, Magnetic field strength, Deep learning, Generative adversarial network, Fully convolutional network

## Abstract

**Background:**

Generative adversarial networks (GAN) can produce images of improved quality but their ability to augment image-based classification is not fully explored. We evaluated if a modified GAN can learn from magnetic resonance imaging (MRI) scans of multiple magnetic field strengths to enhance Alzheimer’s disease (AD) classification performance.

**Methods:**

T1-weighted brain MRI scans from 151 participants of the Alzheimer’s Disease Neuroimaging Initiative (ADNI), who underwent both 1.5-Tesla (1.5-T) and 3-Tesla imaging at the same time were selected to construct a GAN model. This model was trained along with a three-dimensional fully convolutional network (FCN) using the generated images (3T*) as inputs to predict AD status. Quality of the generated images was evaluated using signal to noise ratio (SNR), Blind/Referenceless Image Spatial Quality Evaluator (BRISQUE) and Natural Image Quality Evaluator (NIQE). Cases from the Australian Imaging, Biomarker & Lifestyle Flagship Study of Ageing (AIBL, *n* = 107) and the National Alzheimer’s Coordinating Center (NACC, *n* = 565) were used for model validation.

**Results:**

The 3T*-based FCN classifier performed better than the FCN model trained using the 1.5-T scans. Specifically, the mean area under curve increased from 0.907 to 0.932, from 0.934 to 0.940, and from 0.870 to 0.907 on the ADNI test, AIBL, and NACC datasets, respectively. Additionally, we found that the mean quality of the generated (3T*) images was consistently higher than the 1.5-T images, as measured using SNR, BRISQUE, and NIQE on the validation datasets.

**Conclusion:**

This study demonstrates a proof of principle that GAN frameworks can be constructed to augment AD classification performance and improve image quality.

**Supplementary Information:**

The online version contains supplementary material available at 10.1186/s13195-021-00797-5.

## Introduction

Rapid improvements in neuroimaging techniques such as magnetic resonance imaging (MRI) have led to more sensitive methods of identifying neurodegeneration associated with Alzheimer’s disease (AD) pathology [1]. Evaluation of the pathophysiological changes on MRI could potentially facilitate the discovery of new treatments and help patients, families, and clinicians. Accurate detection of AD using MRI is contingent on the signal-to-noise ratio (SNR) of the scan data, which is directly connected to instrument-related parameters such as magnetic field strength. As such, scanner improvements can lead to increased sensitivity to detect subtle biological changes. Nevertheless, image-based screenings of at-risk individuals are usually carried out by relying on a single scanner technology. This means that the ongoing national studies such as the Alzheimer’s Disease Neuroimaging Initiative (ADNI) [2], the Australian Imaging, Biomarker & Lifestyle Flagship Study of Ageing (AIBL) [3], and the National Alzheimer’s Coordinating Center (NACC) [4], dedicated to detection of AD and tracking AD progression, can allow us to generate AD classification models with accuracies that are bounded by advancements in the scanners.

One possible solution to partially address this issue is using generative adversarial learning [5], which is an emerging technique in machine learning that incorporates a system of two neural networks that compete with each other in a zero-sum game framework. Since its introduction, there has been a surge of interest in the application of GAN frameworks related to the brain. Some of the applications include image generation with improved properties such as achieving super resolution or better quality [6–11], data augmentation [12–14], segmentation [9, 13–16], image reconstruction [17–20], image-to-image translation [21–24], and motion correction [25, 26]. While these important studies have demonstrated the exciting prospect of using GAN architectures, there is a limited amount of work that has focused on utilizing the generated images for subsequent tasks such as disease classification [27]. Here, we evaluated if a generative adversarial network (GAN) can be developed to augment performance of a classifier trained using the generated images. To achieve this goal, we processed brain MRI scans of multiple magnetic field strengths (1.5 Tesla (1.5 T) and 3 Tesla (3 T)) from the ADNI dataset and also obtained access to 1.5-T MRI scans from the AIBL and NACC datasets. Using these data, we addressed the following objective. The deep learning framework needs to more accurately predict the class label than what one could achieve using the original scans. To achieve this, we trained a GAN model using 1.5-T and 3-T scans obtained around the same time on the same set of individuals. Using well-known image quality metrics, we compared the original scans and the generated images. We then used the generated images to construct a fully convolutional network (FCN) that discriminated between cases who had AD from those who had normal cognition. For comparison, we also generated an independent FCN model using the original 1.5-T scans to predict AD status. Validation of the FCN models was performed using data from the AIBL and NACC studies.

## Materials and methods

### Study population and MRI scan parameters

We obtained access to T1-weighted MRI scans from the ADNI (*n* = 417), AIBL (*n* = 107), and NACC (*n* = 565) cohorts (Table 1). For a subset of the ADNI data (*n* = 151), both 1.5-Tesla (1.5-T) and 3-Tesla (3-T) scans taken at the same time were available, and 1.5-T scans were available from the other cohorts. All the MRI scans considered for this study were performed on individuals within ±6 months from the date of clinical assessment.

**Table 1:** Study population and characteristics

	ADNI 1.5 T			ADNI 3 T			NACC		AIBL	
Diagnosis	NC	MCI	AD	NC	MCI	AD	NC	AD	NC	AD
**Number of cases**	229	69	188	47	69	35	356	209	93	14
**Age (median + range)**	76 [60, 90]	76 [55, 88]	76 [55, 91]	75 [70–86]	76 [55, 88]	72 [57, 89]	74 [56, 94]	77 [55, 95]	71 [61, 86]	73 [58, 82]
**Gender, male (percentage)**	119 (51.96%)	39 (56.52%)	101 (53.72%)	18 (38.29%)	39 (56.52%)	12 (34.29%)	126 (35.39%)	95 (45.45%)	48 (51.61%)	6 (42.86%)
**Education (median + range)**	16 [6, 20]	16 [6, 20]	16 [4, 20]	16 [7, 20]	16 [6, 20]	14 [7, 20]	16 [0, 22]	14.5 [2, 24]	N.A.	N.A.
**APOE+ (percentage)**	61 (26.64%)	33 (47.83%)	124 (65.96%)	13 (27.66%)	33 (47.83%)	24 (68.75%)	102 (28.65%)	112 (53.59%)	1 (1.01%)	1 (7.17%)
**MMSE (median + range)**	29 [25, 30]	26 [24, 30]	23.5 [18, 28]	30 [26, 30]	26 [24, 30]	23 [20, 27]	29 [20, 30]	22 [0, 30]	29 [25, 30]	18 [6, 22]

Three independent datasets including (a) the Alzheimer’s Disease Neuroimaging Initiative (ADNI) dataset, (b) the Australian Imaging, Biomarker & Lifestyle Flagship Study of Ageing (AIBL), and (c) the National Alzheimer’s Coordinating Center (NACC) were used for this study

ADNI is a longitudinal multicenter study designed to develop clinical, imaging, genetic, and biochemical biomarkers for the early detection and tracking of AD [28]. AIBL, launched in 2006, is the largest study of its kind in Australia and aims to discover biomarkers, cognitive characteristics, and lifestyle factors that influence the development of symptomatic AD [3]. Finally, NACC, established in 1999, maintains a large relational database of standardized clinical and neuropathological research data collected from AD centers across the USA [29].

The MRI scans used in this study from the ADNI dataset are from the baseline visit. For a subset of the ADNI participants (*n* = 151), both 1.5-T and 3-T scans taken at the same study visit were available. Scanning on ADNI focused on consistent longitudinal structural imaging on 1.5-T scanners using T1-weighted sequences, and a group of subjects were also scanned using the same protocol on 3-T scanners. For each scanning sequence (MP-RAGE), the geometry defining the field of view at reconstructed resolution was 208 × 240 × 256 mm^3^ at 1 × 1 × 1 mm^3^. The timing parameters included TE = min full echo, TR = 2300, and T1 = 900, and the approximate runtime was 6.2 min. These scans from the ADNI data were primarily used for GAN model development. The remaining participants from the ADNI, AIBL, and NACC studies had 1.5-T MRI scans available and were primarily used for FCN model development. Note that the AIBL study also used an imaging protocol similar to the ADNI study. However, for the NACC study, a single protocol was not available as it is a collection of scans from several AD centers.

### Image registration and data normalization

We first applied a data preprocessing pipeline on raw MRI scans. The pipeline consecutively performs linear image registration, intensity normalization, background removal, and outlier clipping on the MRIs, as described below.

We used the linear registration tool from the FSL package (University of Oxford, UK) to register raw MRIs based on the MNI152 template (ICBM 2009c Nonlinear Symmetric template, McGill University, Canada). All the 1.5-T MRIs were registered to the MNI template and the 3-T MRIs available on the same subjects were co-registered with the registered 1.5-T MRIs. Since linear registration was used, the registered images were automatically resized to match the head size of the MNI template. We then applied *z*-score intensity normalization on all the voxels of the whole brain volume so that the mean and the standard deviation values of all voxels within an MRI were 0 and 1, respectively.

We then removed the background noise by setting all background voxels to the value of − 1. A depth-first search (DFS) algorithm was implemented to achieve the goal of finding background regions. The DFS algorithm expanded the search volume from initial corner locations into inner regions until the scalp fat signal was encountered which could be distinguished by its high intensity. A threshold value was used to separate the signal of the scalp fat from the background and consequently prohibited the search region leaking into the brain. Lastly, to eliminate outlier voxels with high intensity, we clipped every voxel to the range [− 1, 2.5], by setting any voxel with intensity lower than − 1 to value − 1, and any voxel with intensity higher than 2.5 to 2.5.

### Deep learning framework

**Fig. 1:**
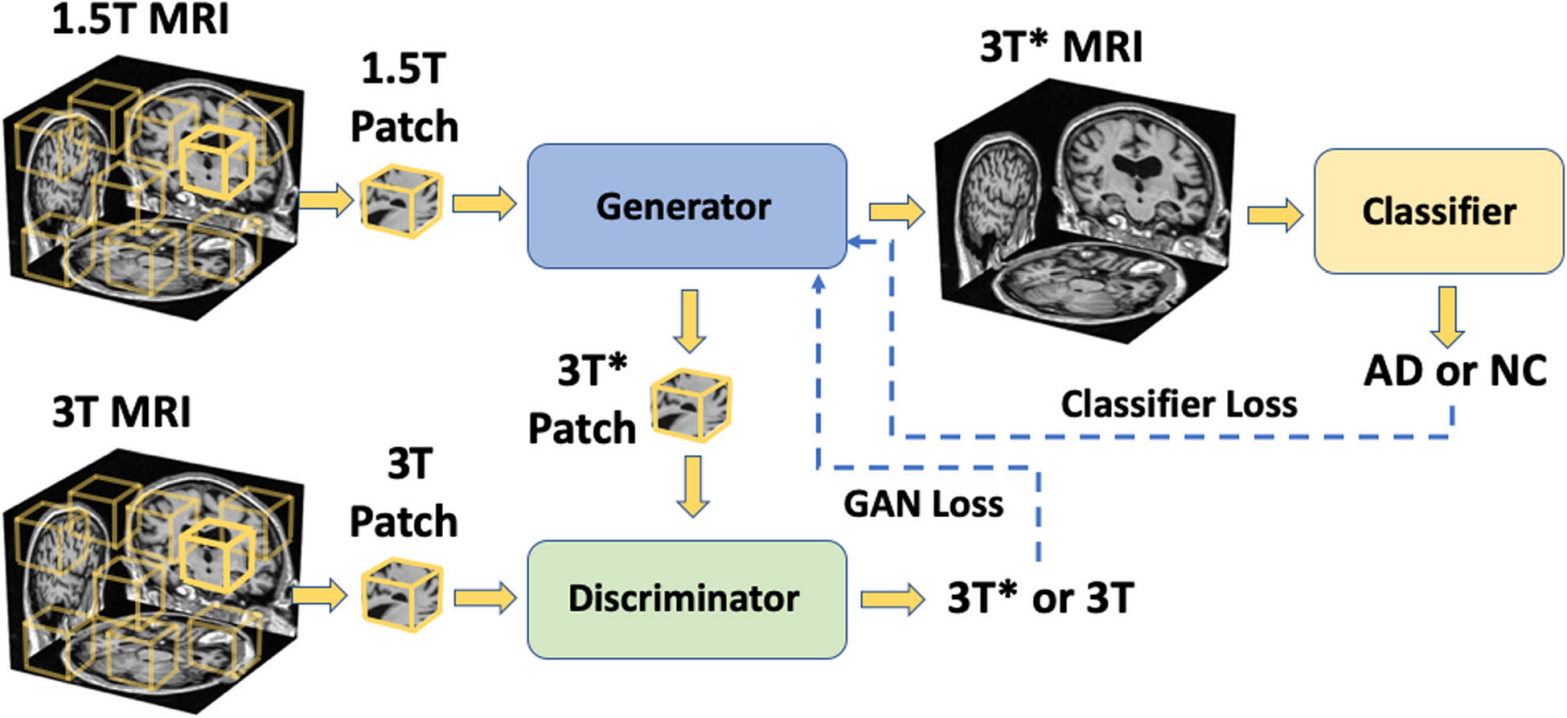
Schematic of the overall deep learning strategy. The generative adversarial network (GAN) uses 1.5-T and 3-T scans of the same individual taken at the same time to generate images (3 T*). The fully convolutional network (FCN) model uses the 3-T* images to predict Alzheimer’s disease (AD) status. Both the GAN and FCN models were trained simultaneously by backpropagating the losses from the GAN and the FCN models

Original 1.5-T scans from the ADNI data on individuals who underwent both 1.5-T and 3-T scanning were fed into the generator that created images, and the discriminator was used to compare the generated images (3T*) with the original 3-T images. To note, the terms “original 1.5T” and “original 3T” refer to the processed original MRIs and the term “generated” images or scans refer to the ones generated by the GAN model. At the same time, a fully convolutional network (FCN) was trained using the generated 3-T* images to discriminate AD cases with the ones who had normal cognition (NC). MRIs from subjects with MCI used for the GAN training were not utilized in the FCN training since our current FCN model only performed binary classification task between AD and NC. The goal was to find a mapping function from 1.5-T to 3-T images for the same subject. The approach to creating this mapping function is similar to residual learning, where the outcome of the generator is a “transformation mask,” which attempts to approximate the difference between 1.5-T and 3-T images. When this mask is added to a 1.5-T scan, it is expected to generate an image (3 T*), which has the same or better image quality and leads to a more accurate prediction of AD status. While the generator is creating a transformation mask, the discriminator is attempting to distinguish between the generated 3-T* image and the original 3-T image in an adversarial fashion. Concomitantly, the FCN model is attempting to distinguish between AD and NC cases. In essence, both the GAN and the FCN model training was performed simultaneously while minimizing the GAN and classifier losses (Fig. 1). This was achieved by allowing the gradient calculated from the FCN classification loss to propagate back to the generator to implicitly convey the disease-related information to the generator. As a result, the classification loss that was propagated provided a momentum for the generator to generate images that contributed to lower cross-entropy loss and thus facilitated better image classification.

The generator of the GAN model consists of three 3D convolutional blocks in which each convolutional operation was followed by batch normalization and rectified linear unit (ReLu) activation. In each convolution layer, the stride and kernel size were set at 1 and 3, respectively, and padding as 2, 0, and 1, to guarantee the output from the generator to have the same size as input so that we could directly add the transformation mask on the 1.5-T scan. The discriminator of the GAN model is fully convolutional, consisted of 5 convolutional blocks in which 3D convolution operations were followed by batch normalization and LeakyReLu activation. The model was trained with losses from the discriminator and the FCN classifier, as well as an additional L_1_-norm loss calculated between the original 3-T image and the generated 3-T* image. More details are described in the supplement. The FCN model was trained to predict AD status using the generated images (3 T*) as inputs. The NINCDS-ADRDA criterion was used to define the AD status [30].

We used patch-wise training for both the GAN and the FCN models. The training process using this strategy was less computationally intensive and allowed us to use neural networks with larger capacity given a total memory budget. Specifically, we randomly sampled patches of size 47 × 47 × 47 from the whole volume as inputs to the deep learning framework. Patches from the 1.5-T scans were sent into the generator, and the discriminator then attempted to differentiate between the 3-T* and 3-T patches. The FCN model then used the 3-T* patches as input to predict AD status. With the strategy of randomly sampling patches over the whole volume, a degree of data augmentation was achieved because the model was trained with more variance of the inputs sampled from various locations. Similar FCN frameworks have been used recently to generate high performance AD classification models [31].

For comparison, we trained another FCN model using 1.5-T scans of the same individuals to predict AD status. We also constructed another deep learning architecture where the GAN model was trained independently by backpropagating just the GAN loss (Figure S1a), and the FCN model was trained by backpropagating just the classifier loss (Figure S1b). Note that even for this case, the GAN model used the 1.5-T and 3-T scans, whereas the FCN model used the generated images from the GAN as inputs to predict the AD status. For the sake of presentation, we denote this GAN model as simpleGAN and the FCN model as simpleFCN.

### Quality metrics

We used signal to noise ratio (SNR) as well as no-reference algorithms including Blind/Referenceless Image Spatial Quality Evaluator (BRISQUE) [32], and Natural Image Quality Evaluator (NIQE) [33], to compare the differences between the original scans and generated images. When evaluating the image quality, we retrieved the center slice in each scan within the brain and then calculated average over this slice for each case.

For SNR, we computed the average values of pixel intensity and divided it by its standard deviation. BRISQUE focuses on quantifying spatial distortion, such as ringing, blur, or blocking, from natural images. Certain regular statistical properties of natural images could be influenced by the presence of distortions. The BRISQUE evaluator was developed by learning the difference between original natural images and distorted images using the LIVE IQA database [34, 35]. NIQE is also a no-reference evaluator, which quantifies image quality according to the level of distortions. The difference of NIQE compared with BRISQUE is that NIQE does not require distorted images as a prior and thus could learn only from undistorted images. Lower BRISQUE and NIQE scores indicate better image quality.

### Data partitioning and computing infrastructure

The models (GAN and simpleGAN) were constructed on a subgroup of ADNI data (*n* = 151), which contained both 1.5-T and 3-T scans from the same individuals taken at the same time. This subgroup was randomly split into training, validation, and testing in the ratio of 3:1:1 (Figure S2). The GAN models were constructed on the training part (60%) of the 151 cases, and they were saved at the instance when SNR on the validation part of the data (20%) was the highest. Image quality of the generated 3-T* images was evaluated on the remaining 20% of the subgroup as well as on the remaining cases in the ADNI data and two external datasets (AIBL & NACC) (Table 1). We must note that the GAN and FCN models were constructed simultaneously. During the GAN model training, the generated 3-T* patches sampled from AD and NC cases were fed into the FCN model to perform AD versus NC classification. Considering the limited number of AD and NC cases from the subgroup who went through both 1.5-T and 3-T MRI scans, we combined all the NC and AD cases from this subgroup along with a randomly selected subgroup from the remaining AD and NC subjects from the ADNI dataset to train the FCN model. In total, 251 subjects were used for FCN model training. The remaining AD and NC subjects from the ADNI dataset (*n* = 166) were randomly and equally split for FCN model validation and testing. The FCN model was saved when the classification accuracy was highest on the validation dataset. The classification performance of the trained FCN model was evaluated on the ADNI testing subset and two external datasets (AIBL and NACC) (Table 1). For comparison, we also trained a separate FCN model using the corresponding 1.5-T scans to predict AD status. Specifically, the cases used for training, validation, and testing of the 1.5-T-based FCN model were the same as those used for the FCN model based on the 3-T* images.

We developed the deep learning framework using PyTorch 1.7.0. Model development was performed on a computing workstation containing a GeForce RTX 2080 Ti (NVIDIA, Santa Clara, CA) GPU card with 11 Gb memory. Model training took approximately 9 h and testing on a new case was almost an instantaneous process taking a few seconds on the same workstation.

### Performance metrics for classification

We generated sensitivity-specificity (SS) and precision-recall (PR) curves based on model predictions on the ADNI test data as well as on the other independent datasets (AIBL and NACC). For each SS and PR curve, we computed the area under curve (AUC) values. Additionally, we computed sensitivity, specificity, F1-score, and Matthews correlation coefficient (MCC) on each set of model predictions. Both the 1.5 T- and 3 T*-based classification models were trained 25 times with various random seeds and 95% confidence intervals were generated.

### Statistical analysis

To evaluate the mean difference in image quality generated by the GAN model, we performed analysis of variance (ANOVA) on the ADNI test data. Image quality was assessed using SNR, BRISQUE, and NIQE. Specific group differences between 1.5-T, 3-T, and 3-T* images were evaluated using the post hoc Tukey test (Table S1). The effect of age, education, gender, MMSE scores, ApoE4 status, and type of scanner on SNR, BRISQUE and NIQE were evaluated using a stepwise forward selection process using the “GLMSELECT” function (SAS Software) followed by analysis of covariance (ANCOVA) (Table S2). Lastly, we used the *t*-test to assess whether the mean image quality of the 1.5-T group was different from the 3-T* group in the NACC and AIBL datasets.

## Results

**Fig. 2:**
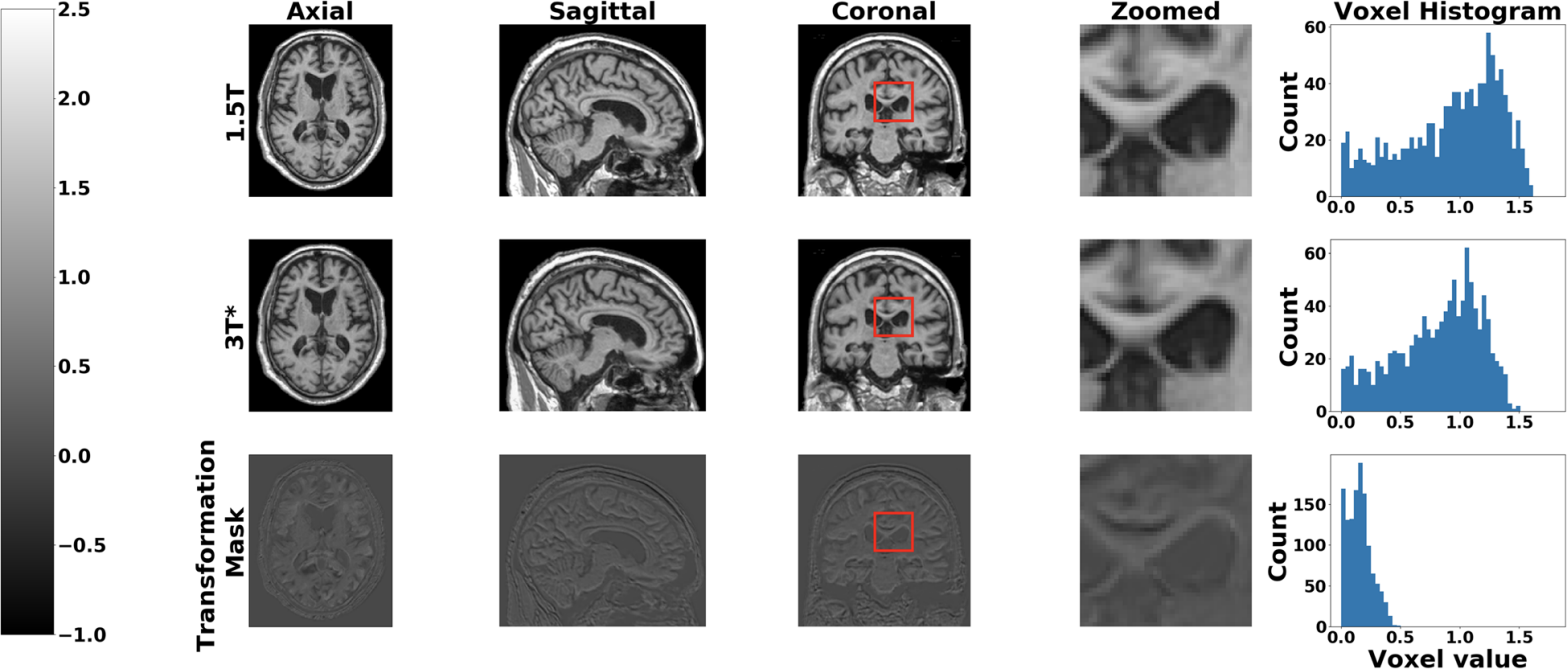
Original and generated images and corresponding transformation masks. Axial, sagittal, and coronal views of a single subject are shown in the first, second, and third columns, respectively. The first row corresponds to the original 1.5-T slices, the second row corresponds to the generated images (3 T*), and the third row corresponds to the difference between 1.5-T and 3-T* images, denoted as the transformation mask. We also showed the same zoomed-in region from 1.5-T and 3-T* images and the transformation mask in the fourth column to reveal the difference between 1.5-T and 3-T* images. Additionally, we presented histograms of the voxel values within the zoomed-in region in the fifth column

Volumetric patch-level training on the 1.5-T and 3-T scans allowed training of volumetric transformation mask, which then resulted in the generation of 3-T* volumetric images. For ease of visualization, we selected the center slice of a single subject and retrieved a two-dimensional transformation mask from the learned volume and compared both the original and generated images (Fig. 2). This figure demonstrates that captured differences between the 1.5-T and 3-T* images were subtle and distributed throughout the region. When metrics such as SNR were used, there was a 9.6% improvement (1.31 to 1.45), on the mean image quality on the ADNI test data between the 1.5-T and 3-T* images. We also found that mean SNR improved by about 11.1% (1.26 to 1.40) and 11.7% (1.28 to 1.43) on the AIBL and NACC data, respectively.

Absolute measures of image quality using perceptual quality metrics provided more insight on the differences between 1.5-T and 3-T* images. When BRISQUE metric was used, there was about 8.3% improvement in the mean image quality on the ADNI test data (49.06 to 44.97), about 10.0% improvement in the mean image quality on the AIBL data (44.91 to 40.44), and about 9.0% mean image quality improvement on the NACC data (47.79 to 43.48). Note that lower BRISQUE score indicates better quality. When NIQE metric was used to compare images, there was 16.8% improvement in the mean image quality on the ADNI test data (7.33 to 6.1), 14.3% improvement in the mean image quality on the AIBL data (7.21 to 6.18), and 16.3% mean image quality improvement on the NACC data (7.99 to 6.69). Similar to the BRISQUE metric, lower NIQE score indicates better quality. These no-reference metrics provided an objective way to evaluate the quality of the generated images on different cohorts (AIBL and NACC), which then grounded our hypothesis that the GAN model can learn from images of multiple magnetic field strengths to improve image quality.

**Fig. 3:**
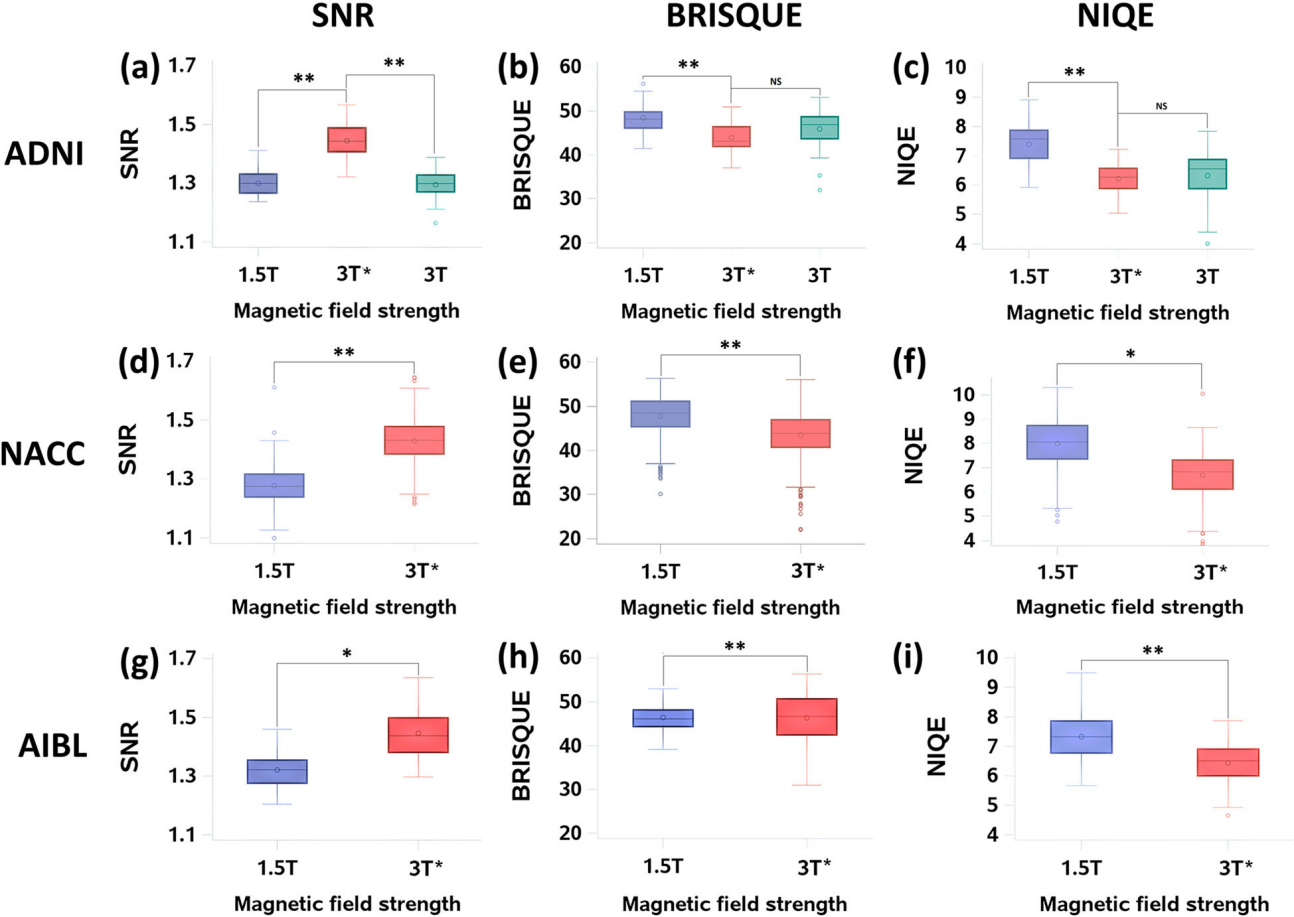
Image quality analysis. Metrics such as SNR as well as no-reference algorithms including BRISQUE and NIQE were used to evaluate the quality of the generated images (3 T*) and compare them with the quality of the original scans (1.5 T and 3 T). The metrics were computed independently on the MRI scans from each study cohort (ADNI-test (**a**–**c**), NACC (**d**–**f**), AIBL (**g**–**i**)). Lower value of the metrics indicates improved quality. The symbol “*” indicates *p* < 0.001 and “**” indicates *p* < 0.0001

In order to determine if there was an overall difference in the mean quality of images produced by the GAN model, we performed ANOVA for the SNR, BRISQUE, and NIQE metrics computed on the ADNI test data (Fig. 3 and Figure S3). We found a significant overall difference in image quality between the 1.5-T, 3-T, and 3-T* groups using SNR (*F* = 229.66, *p* < 0.0001), BRISQUE (*F* = 10.80, *p* < 0.0001), and NIQE (*F* = 27.95, *p* < 0.0001). To identify the between-group differences for the SNR, BRISQUE, and NIQE metrics, we used the Tukey’s post hoc procedure (Table S1). We found that the 3-T* group had significantly better image quality compared to 1.5-T scans across all three image quality metrics, SNR (*p* < 0.0001), BRISQUE (*p* < 0.0001), and NIQE (*p* < 0.0001). On the other hand, the mean image quality in the 3 T* category was significantly better than the 3-T scans on the SNR metric (*p* < 0.0001), but not on the BRISQUE (*p* = 0.1074) and NIQE (*p* = 0.82) metrics.

A stepwise forward selection process using the “GLMSELECT” function (SAS Software) was used to evaluate the effect of age, education, gender, MMSE scores, ApoE4 status, and type of scanner on SNR, BRISQUE, and NIQE (Table S2). For SNR, “age” was statistically significant (*p* = 0.0003). For BRISQUE, “years of education” (*p* = 0.04) and “scanner type” (*p* = 0.003) were statistically significant, and for NIQE, “age” (*p* = 0.001), “years of education” (*p* = 0.01), and “scanner type” (*p* = 0.006) were statistically significant. Lastly, an analysis of covariance was performed by adjusting for the abovementioned covariates. The mean difference in image quality assessed by SNR in both the 1.5-T and 3-T groups was 0.15 units lower than the 3-T* scans (*p* < 0.0001) after adjusting for age. After adjusting for years of education, the mean difference in image quality between 1.5-T and 3-T* images assessed by the BRISQUE metric was 4.53 (*p* < 0.0001) and between 3-T* and 3-T images was 2.0 (*p* = 0.04). Lastly, after adjusting for age, years of education, and type of scanner, the mean difference in image quality assessed by NIQE between 1.5-T and 3-T* images was 1.16 units (*p* < 0.0001); however, the association was not significant between 3-T* and 3-T images (*p* = 0.54).

**Fig. 4:**
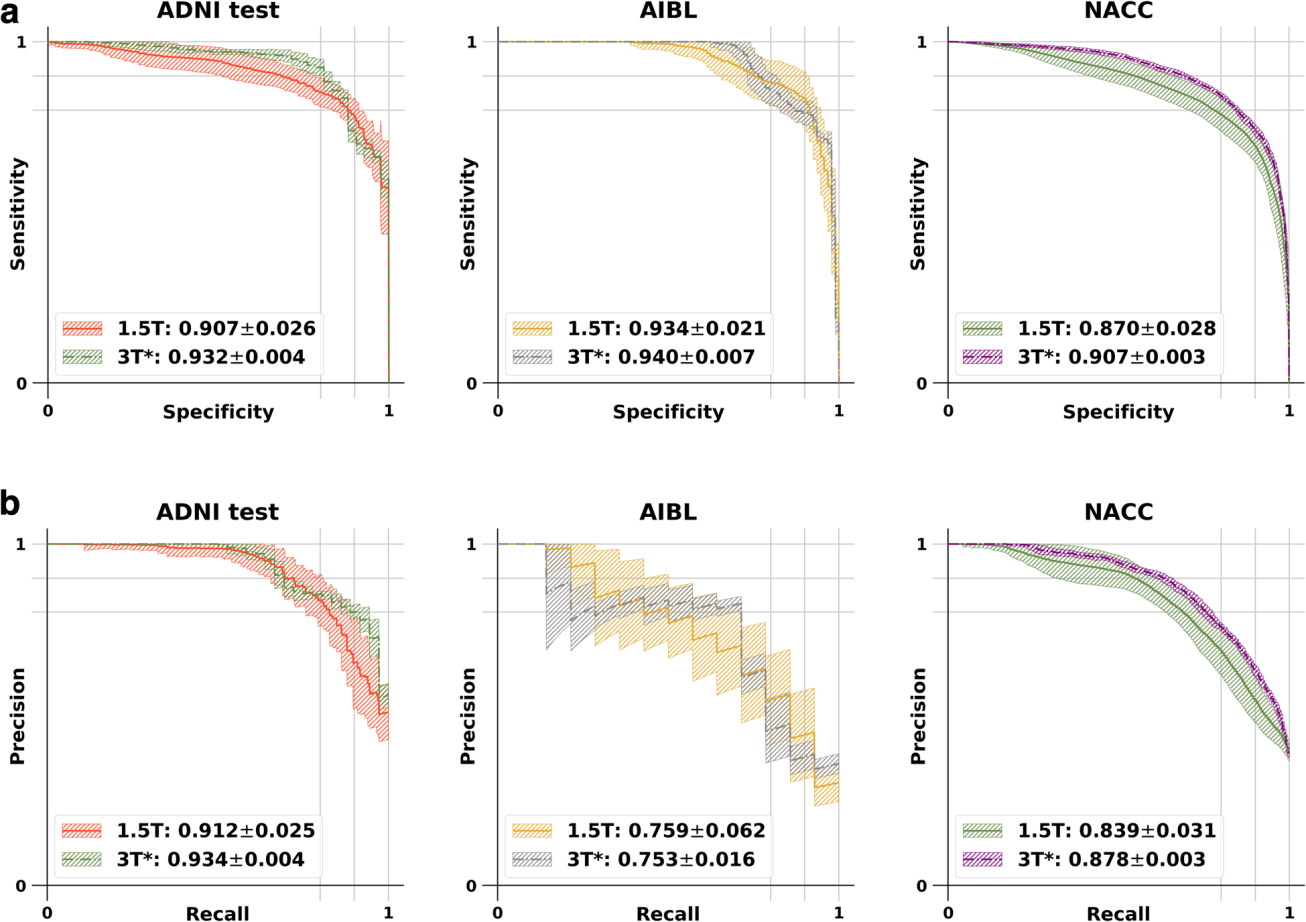
Performance of the FCN models. **a** Sensitivity-specificity (SS) and **b** precision-recall (PR) curves comparing the FCN models predicting AD status. One FCN model was developed using the 1.5-T scans and the other using the 3-T* images. Model performance is shown on all three datasets (ADNI test, AIBL, and NACC)

The generated images led to consistent, high AD classification performance across the external datasets, at least as demonstrated by area under the sensitivity-specificity and the precision-recall curves (Fig. 4a, b). The FCN model based on the 3-T* images demonstrated improved performance on both the AIBL and NACC datasets, using most of the computed performance metrics (Table 2a and b). It is worth noting that for MCC, which is generally regarded as a balanced measure and can be used even if the classes are of different sizes, the mean MCC value increased by 19.6% on the AIBL dataset (0.5757 to 0.6884) and 9.2% on the NACC dataset (0.6032 to 0.6585), respectively. F1-score, which is generally used as a weighted score of the model’s performance, also increased by 18.8% on the AIBL dataset (0.6126 to 0.7276) and 6.4% on the NACC data (0.7301 to 0.7768). The 95% confidence intervals for the FCN models show that the model predictions were fairly consistent across different runs and varied between the FCN models based on 1.5-T and 3-T* images, respectively (Table 3). Of note, the FCN model based on the 3-T* images performed better than the simpleFCN on the ADNI test and NACC datasets and was relatively similar on the AIBL dataset, as demonstrated by the area under the SS and PR curves (Figure S4a & b). In general, the simpleGAN-based 3-T* images had improved quality compared to 1.5-T images (Figures S5 & S6, Table S3). More details on these findings can be found in the supplement.

**Table 2:** Performance of the FCN models

(a)					
**1.5 T**	**Accuracy**	**Sensitivity**	**Specificity**	**F-1**	**MCC**
**ADNI test**	0.8398 ± 0.0238	0.7363 ± 0.0514	0.9209 ± 0.0385	0.7972 ± 0.0325	0.6766 ± 0.0492
**AIBL**	0.8873 ± 0.0647	0.6309 ± 0.1393	0.9259 ± 0.0874	0.6126 ± 0.1016	0.5757 ± 0.1086
**NACC**	0.8157 ± 0.0224	0.6739 ± 0.0605	0.8989 ± 0.0616	0.7301 ± 0.0221	0.6032 ± 0.0425
(b)					
3T*	**Accuracy**	**Sensitivity**	**Specificity**	**F-1**	**MCC**
**ADNI test**	0.8210 ± 0.0143	0.7411 ± 0.0312	0.8895 ± 0.0114	0.7923 ± 0.0195	0.6416 ± 0.0280
**AIBL**	0.9293 ± 0.0132	0.7143 ± 0.0000	0.9617 ± 0.0152	0.7276 ± 0.0365	0.6884 ± 0.0449
**NACC**	0.8429 ± 0.0069	0.7393 ± 0.0180	0.9037 ± 0.0134	0.7768 ± 0.0096	0.6585 ± 0.0149

Accuracy, sensitivity, specificity, F1-score, and Matthew’s correlation coefficient are computed for the FCN models that used (a) 1.5-T scans and (b) 3-T* images, respectively

**Table 3:** Confidence intervals of model performance

	1.5 T	3 T*	3 T* − 1.5 T
(a)			
**ADNI test**	[0.8968, 0.9172]	[0.9304, 0.9336]	[0.0203, 0.0297]
**AIBL**	[0.9258, 0.9422]	[0.9373, 0.9427]	[0.0021, 0.0097]
**NACC**	[0.8590, 0.8810]	[0.9058, 0.9082]	[0.0314, 0.0412]
(b)			
**ADNI test**	[0.9022, 0.9218]	[0.9324, 0.9356]	[0.0175, 0.0265]
**AIBL**	[0.7347, 0.7833]	[0.7467, 0.7593]	[−0.0170, 0.0051]
**NACC**	[0.8268, 0.8512]	[0.8768, 0.8792]	[0.0334, 0.0443]

(a) 95% confidence intervals of the SS curves for 1.5-T-based model, 3-T*-based model, and difference of AUCs between 3-T* and 1.5-T-based models. (b) 95% confidence intervals of PR curves for 1.5-T-based model, 3-T*-based model and difference of AUCs between 3-T* and 1.5-T-based models

## Discussion

Our deep learning pipeline involved training of a GAN model to learn from 1.5-T and 3-T scans obtained from the same subjects at the same time, and an FCN model to simultaneously predict AD status that used the generated 3-T* images from the GAN model. Simultaneous minimization of losses from the GAN and the FCN models enabled us to achieve improvements in MR image quality and AD classification performance. Moreover, volumetric patch-level training of the GAN and the FCN models turned out to be computationally efficient, where the size of the patches was the same as the receptive field of the FCN model. Importantly, access to 1.5-T and 3-T scans on the same subjects taken at the same time was crucial to develop 3-T* images, without the influence of potential confounding factors such as scan timing. Also, both the AIBL and NACC datasets served as good, independent datasets for model validation, allowing use of a similar criterion for subject selection on these cohorts.

There is a putative link between MRI scans with high quality (defined using SNR, etc.) obtained from latest instrument-level advancements and their ability to better delineate structural aspects that manifest in various diseases. It is appealing to embrace MR images of high SNR to improve detection of structural changes in the human brain. This seemingly advantageous technological progress poses a conundrum—models created using MR images at early time points using older technology may not be sufficiently accurate in terms of predicting AD status. Further, longitudinal changes determined from 1.5-T and 3-T scans due to neurodegeneration cannot be confounded by increased sensitivity due to higher magnet strength. This becomes more important in the case of aging individuals who could benefit from a more accurate assessment of cognitive status early in their lives. While there is not yet any available drug treatment for treating cognitive abnormalities with insidious onset such as AD, research indicates that delaying onset will cut an individual’s risk for diagnosis [36, 37]. Using the GAN framework and MR images of different MFS, we developed a model to generate images of improved quality and predict AD status of individuals with greater accuracy.

### Study limitations

Our study has a few limitations. First, the sample size used for GAN model training was small (*n* = 151), as only a limited number of cases had both 1.5-T and 3-T imaging done at the same time. It is possible to generate a more robust GAN model if such data is available on a larger number of cases. Both the GAN and FCN models were designed to have specific architectures. More optimized architectures can be constructed and this could alter the performance of the models. We used well-known no-reference algorithms to evaluate image quality on the images, and additional quality metrics could be explored. Even though the BRISQUE evaluator was not designed to evaluate medical images, we still used it to explore whether distortion features from natural images could statistically distinguish any subtle differences between MRIs collected with various magnetic field strengths. We observed that the image quality of the generated 3-T* scans statistically outperformed that of the original 1.5-T scans. Nevertheless, the ability of the GAN model to generate images of similar quality to that of the original scans was consistent across these metrics, and the enhanced AD classification performance was evident, as evaluated using independent test data. In future, we will expand our classification task to include MCI cases and further stratify MCI subjects into those who remain stable from the ones who convert to dementia.

## Conclusions

Our approach to produce high AD classification performance models using a deep learning framework could transform the way MRI scans are utilized in AD research. Our study implication is that it is possible to generate images of enhanced quality on disease cohorts that have previously used the 1.5-T scanners, and in those centers who continue to rely on 1.5-T scanners. This would allow us to reconstruct the earliest phases of AD, and build a more accurate model of predicting cognitive status than would otherwise be possible using data from 1.5-T scanners alone. Our proposed deep learning framework can also be extended to process other medical imaging datasets and organ systems when relevant data is available for model development.

## Supplementary Information

**Additional file 1: Figure S1.** (A) Simple GAN architecture with a generator and a discriminator. (B) Simple FCN classification architecture for prediction of AD status. **Figure S2.** Number of cases from the ADNI cohort used for GAN and FCN model development. The cases covered by the black arrow indicate the ones used for the GAN model development and the cases covered by the red arrow indicate the ones used for the FCN model development. **Figure S3.** ANCOVA analysis to assess the mean image quality using SNR, BRISQUE and NIQE in the ADNI-training (a-c) and ADNI-validation (d-f) data. **Table S1.** Specific group differences in magnetic field strength (1.5T, 3T, 3T*) evaluated using Tukey's post hoc procedure across the image quality measures SNR, BRISQUE and NIQE in the ADNI-training, testing and validation data. **Table S2.** ANOVA analysis of AUCs from the ROC curves on the gender, age and scanner variables to demonstrate whether the AD classification performance differed on various groups. **Figure S4.** Performance of the FCN classifier based on the images generated using the simpleGAN architecture (Figure S1). (A) Sensitivity-specificity and (B) precision-recall curves are shown on the ADNI test, AIBL and NACC datasets, respectively. **Figure S5.** ANCOVA analysis to assess the mean image quality using SNR, BRISQUE and NIQE for simpleGAN model in the ADNI-test (a-c) and t-test results of NACC (d-f) and AIBL (g-i) data, respectively. **Figure S6.** ANCOVA analysis to assess the mean image quality using SNR, BRISQUE and NIQE for simpleGAN model in the ADNI-training (a-c) and ADNI-validation (d-f) data. The effect of independent variables such as age, education, MMSE scores, and type of scanner was evaluated using a stepwise forward selection process and models were adjusted accordingly. **Table S3.** Specific group differences in magnetic field strength (1.5T, 3T, 3T*) were evaluated using Tukey's post hoc procedure across the image quality measures SNR, BRISQUE and NIQE for the simpleGAN model in the ADNI-training, testing and validation data.

## Data Availability

Python scripts and sample data are made available on GitHub (https://github.com/vkola-lab/azrt2020).
