# Supplementary material for: Enhancing magnetic resonance imaging-driven Alzheimer’s disease classification performance using generative adversarial learning
Source: Alzheimers Res Ther. 2021 Mar 14;13:60. doi: 10.1186/s13195-021-00797-5 (PMC7958452; doi:10.1186/s13195-021-00797-5)
Supplement: Supplementary file 1 — Additional file 1: Figure S1. (A) Simple GAN architecture with a generator and a discriminator. (B) Simple FCN classification architecture for prediction of AD status. Figure S2. Number of cases from the ADNI cohort used for GAN and FCN model development. The cases covered by the black arrow indicate the ones used for the GAN model development and the cases covered by the red arrow indicate the ones used for the FCN model development. Figure S3. ANCOVA analysis to assess the mean image quality using SNR, BRISQUE and NIQE in the ADNI-training (a-c) and ADNI-validation (d-f) data. Table S1. Specific group differences in magnetic field strength (1.5T, 3T, 3T*) evaluated using Tukey's post hoc procedure across the image quality measures SNR, BRISQUE and NIQE in the ADNI-training, testing and validation data. Table S2. ANOVA analysis of AUCs from the ROC curves on the gender, age and scanner variables to demonstrate whether the AD classification performance differed on various groups. Figure S4. Performance of the FCN classifier based on the images generated using the simpleGAN architecture (Figure S1). (A) Sensitivity-specificity and (B) precision-recall curves are shown on the ADNI test, AIBL and NACC datasets, respectively. Figure S5. ANCOVA analysis to assess the mean image quality using SNR, BRISQUE and NIQE for simpleGAN model in the ADNI-test (a-c) and t-test results of NACC (d-f) and AIBL (g-i) data, respectively. Figure S6. ANCOVA analysis to assess the mean image quality using SNR, BRISQUE and NIQE for simpleGAN model in the ADNI-training (a-c) and ADNI-validation (d-f) data. The effect of independent variables such as age, education, MMSE scores, and type of scanner was evaluated using a stepwise forward selection process and models were adjusted accordingly. Table S3. Specific group differences in magnetic field strength (1.5T, 3T, 3T*) were evaluated using Tukey's post hoc procedure across the image quality measures SNR, BRISQUE and NIQE for the simpleGAN mo [file 13195_2021_797_MOESM1_ESM.docx]

**Enhancing magnetic resonance imaging driven Alzheimer’s disease classification performance using generative adversarial learning**

Xiao Zhou^1,2,*^, Shangran Qiu^1,3,*^, Prajakta S. Joshi^4,5^, Chonghua Xue^1^, Ronald J. Killiany^4,6,7,8^, Asim Mian^6^, Sang P. Chin^2,9,10^, Rhoda Au^4,7,8,11,12^, Vijaya B. Kolachalama^1,2,8,13^

**Supplemental information**

**Data partitioning**

The ADNI dataset was partitioned based on the number of 1.5T and 3T scans that were made available in the public domain. For the GAN model development, 47 NC, 69 MCI and 35 AD cases were used and for the FCN model development, 229 NC cases (182+47) and 188 AD cases (153+35) were used. Both 1.5T and 3T scans were available on 47 NC, 69 MCI and 35 AD subjects. The 1.5T scans were available on all the cases. Note that the 47 NC and 35 AD cases were used for both GAN and FCN model development.

**(A)**


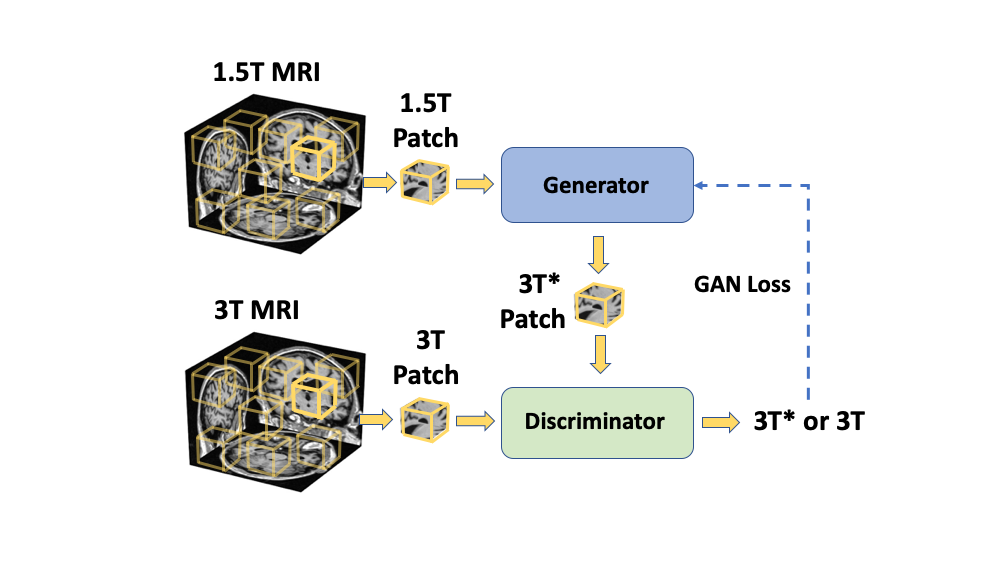


**(B)**


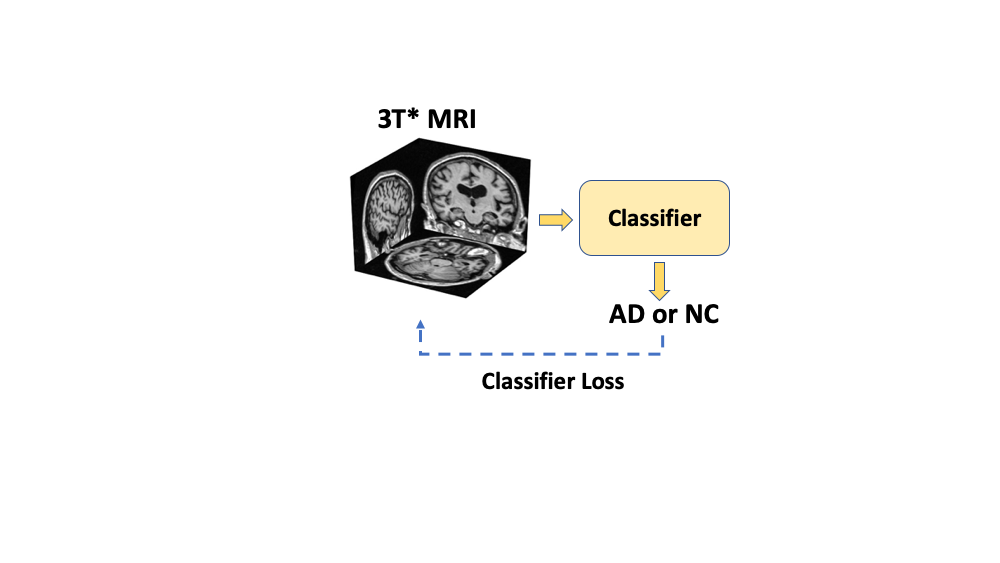


Figure S1: (A) Simple GAN architecture with a generator and a discriminator. (B) Simple FCN classification architecture for prediction of AD status.

**
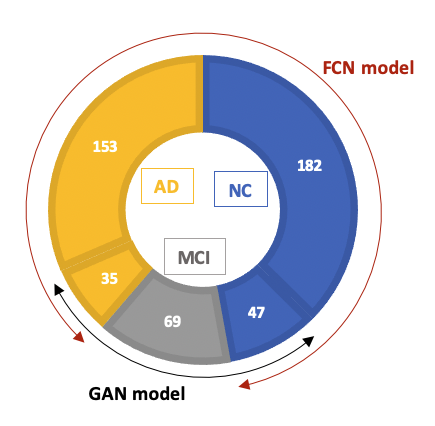
**

Figure S2: Number of cases from the ADNI cohort used for GAN and FCN model development. The cases covered by the black arrow indicate the ones used for the GAN model development and the cases covered by the red arrow indicate the ones used for the FCN model development.

**Generative adversarial framework**

The framework consists of three models, i.e., generator, discriminator and a disease classifier. The generator takes 1.5T MRI as input and produces a scan where we call enhanced 3T* MRI. The discriminator tries to distinguish the generated 3T* scan from 3T scan and sends difference information back to the generator. Thus, by performing the two-player game between generator and discriminator, generator is able to produce more and more realistic scans with the aid from discriminator and at the meanwhile, discriminator also got improved in classifying the subtle difference between the generated 3T* and real 3T scans. The loss functions for the discriminator is the binary cross entropy loss as shown below:

$$lossD=-log\left( D\left( x \right) \right)-log\left( 1-D\left( G\left( z \right) \right) \right)$$

where x represents real 3T scans, z represents 1.5T scan, G and D refer to the generator and discriminator models, respectively. Note that G(z) is the generated 3T* scan. In this setting, the discriminator is trained by assigning 3T as real images and 3T* as fake images.

The generator is supposed to generate scans which discriminator might consider as real scans, thus the first part of the loss function of the generator is:

$$lossG1=-log\left( D\left( G\left( z \right) \right) \right)$$

Since we trained the GAN model by sending in paired 1.5T and 3T scans from same subjects, the generated images should be ideally the same as 3T scans. Thus, the second part of generator’s loss function is L1 norm between generated 3T* and 3T scans:

$$lossG2=\left| \left( G\left( z \right)-x \right) \right|_{1}$$

Besides these two parts, the GAN framework was trained together with an auxiliary disease classifier, which is another fully convolutional network, denoted as FCN. The FCN classifies generated 3T* scans according to the disease label, which is either AD, representing Alzheimer’s disease, or NC, representing normal cognition. The disease classification loss will be back-propagated to the generator as well, which contributes to the third part of the generator’s loss function:

$$lossG3=BCE(y, FCN(G(z)))$$

where BCE represents binary cross entropy loss and the disease ground truth y take values below:

$$y= \left\{ \begin{aligned} 1 if AD \\ 0 if NC \end{aligned} \right.$$

Combining all the three terms, the total loss of the generator is:

$$lossG=-log\left( D\left( G\left( z \right) \right) \right)-\left| \left( G\left( z \right)-x \right) \right|_{1}+BCE(y, FCN(G(z)))$$

Note that while the generator and discriminator training, the FCN was also trained from scratch by learning the same BCE loss below:

$$lossFCN =BCE\left( y, FCN\left( G\left( z \right) \right) \right)=-y\cdot log\left( FCN\left( G\left( z \right) \right) \right) -\left( 1-y \right)\cdot log\left( 1-FCN\left( G\left( z \right) \right) \right)$$

By learning from the generated scans which own large variance due to the stochastic nature of the deep learning generator, FCN can benefit from the data variety as a form of efficient GAN-based data augmentation.


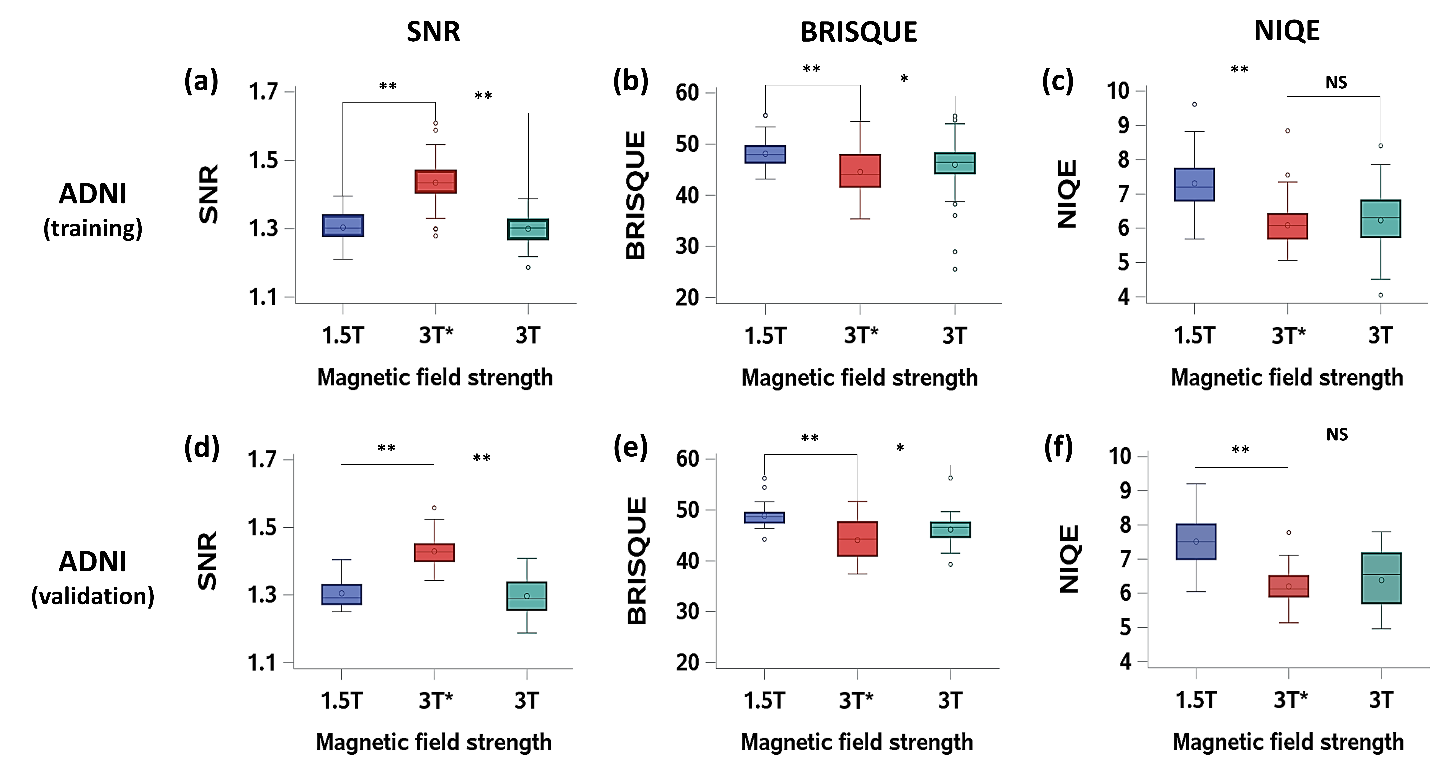


*: Statistical significance at p<0.05 level

**: Statistical significance at p<0.0001 level

Figure S3: ANCOVA analysis to assess the mean image quality using SNR, BRISQUE and NIQE in the ADNI-training (a-c) and ADNI-validation (d-f) data.

In order to determine whether mean quality of images in the ADNI-test and ADNI-validation data differed across the different magnetic field strengths (1.5T, 3T and 3T*), we performed the ANCOVA analysis (Figure S2). Image quality measures included SNR, BRISQUE and NIQE. We found significant evidence of an overall difference in mean image quality using all three measures between 1.5T, 3T and 3T* scans at 0.05 level of significance.

| **ADNI-training** | | | **ADNI-validation** | | **ADNI-testing** | |
| --- | --- | --- | --- | --- | --- | --- |
| **SNR** | | | | | | |
|  | **1.5T** | **3T** | **1.5T** | **3T** | **1.5T** | **3T** |
| **3T*** | <.0001 | <.0001 | <.0001 | <.0001 | <.0001 | <.0001 |
| **3T** | 0.89 |  | 0.79 |  | 0.92 |  |
| **BRISQUE** | | | | | | |
|  | **1.5T** | **3T** | **1.5T** | **3T** | **1.5T** | **3T** |
| **3T*** | <.0001 | 0.04 | <.0001 | 0.02 | <.0001 | 0.09 |
| **3T** | 0.0006 |  | 0.002 |  | 0.02 |  |
| **NIQE** | | | | | | |
|  | **1.5T** | **3T** | **1.5T** | **3T** | **1.5T** | **3T** |
| **3T*** | <.0001 | 0.35 | <.0001 | 0.57 | <.0001 | 0.82 |
| **3T** | <.0001 |  | <.0001 |  | <.0001 |  |

Table S1: Specific group differences in magnetic field strength (1.5T, 3T, 3T*) evaluated using Tukey's post hoc procedure across the image quality measures SNR, BRISQUE and NIQE in the ADNI-training, testing and validation data.

In order to identify specific between group differences in magnetic field strength, we used Tukey's post hoc procedure (Table S1). We evaluated the between group differences in the ADNI data across the training, validation and testing splits. First, we assessed whether the mean image quality of the 3T* and 3T scans was better than the 1.5T scans. We found that the 3T* group had a significantly better mean image quality (p <.0001) than 1.5T scans measured using SNR, BRISQUE and NIQE across the ADNI training, validation and testing data. On the other hand, the mean image quality of 3T scans was only significantly better than the 1.5T scans on the BRISQUE and NIQE measures, but was not significant using the SNR measure in any of the ADNI data splits.

Next, we assessed the whether the mean image quality of the 3T* scans was better than the 3T scans (Table S1). We found that the mean image quality measured using SNR in the 3T* category was significantly better than the 3T scans across all three data splits at <.0001 level of significance. Using the BRISQUE measure, we found that the mean image quality in the 3T* category was significantly better than the 3T scans at 0.05 level of significant in the ADNI training (p=0.04) and validation data (p=0.02), but not in the ADNI test data (p=0.09). However, the mean image quality in the 3T* category was not significantly better than the 3T scans in any of the ADNI data splits, using the NIQE image quality measure.

|  | **F-value** | **P-value** |
| --- | --- | --- |
| **Gender (2 groups)** | 553.525 | 5.29738e-28 |
| **Age (3 groups, low, mid, high)** | 55.3377 | 2.7764e-15 |
| **Scanner (3 groups, Philips, GE, Siemens)** | 367.705 | 1.6165e-38 |

Table S2: ANCOVA analysis of AUCs from the ROC curves on the gender, age and scanners variable to demonstrate whether the AD classification performance differ on various groups.

**(A)**


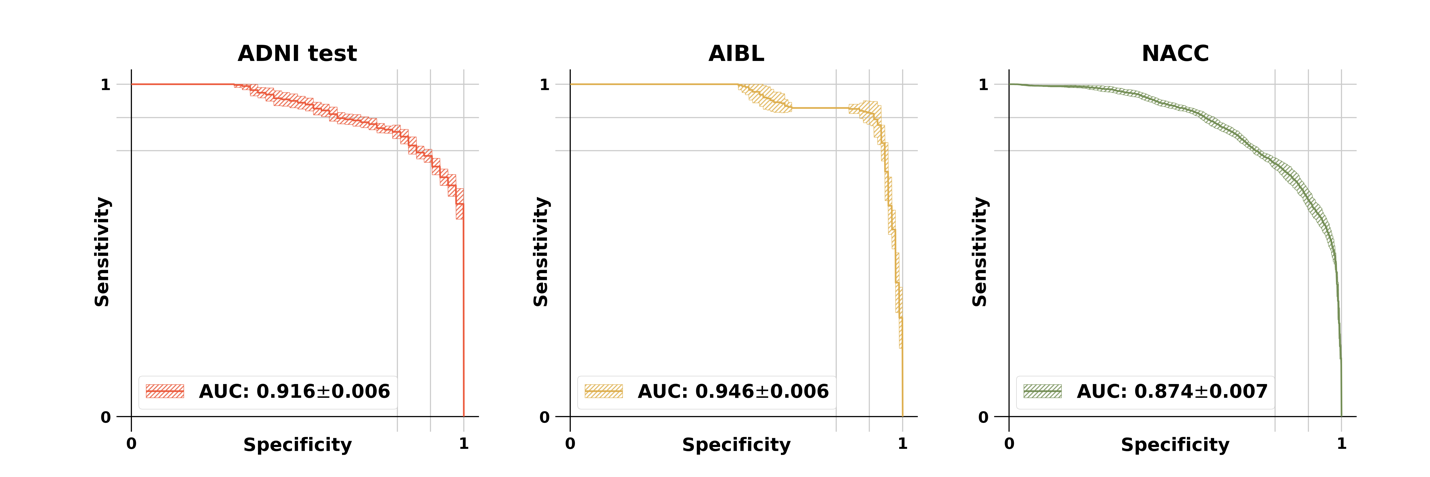


**(B)**


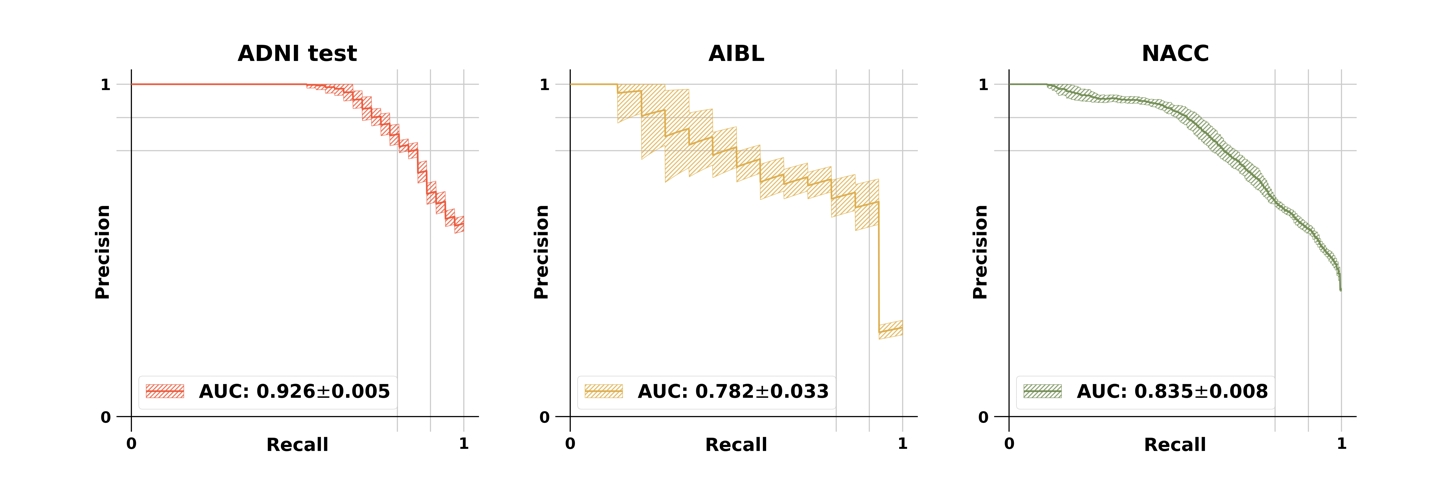


Figure S4: Performance of the FCN classifier based on the images generated using the simpleGAN architecture (Figure S1). (A) Sensitivity-specificity and (B) precision-recall curves are shown on the ADNI test, AIBL and NACC datasets, respectively.


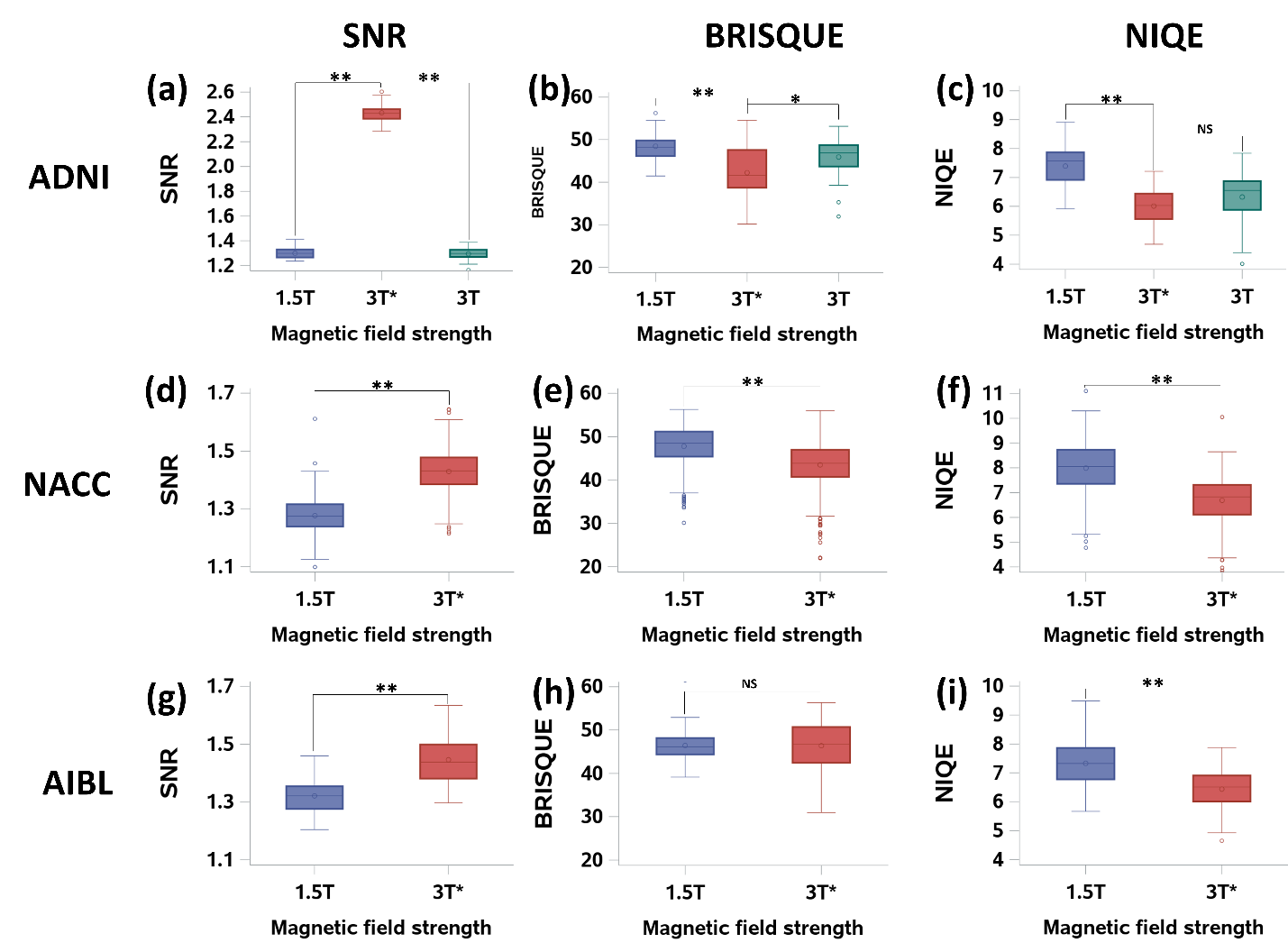


*: Statistical significance at p<0.05 level

**: Statistical significance at p<0.0001 level

Figure S5: ANCOVA analysis to assess the mean image quality using SNR, BRISQUE and NIQE for simpleGAN model in the ADNI-test (a-c) and T-test results of NACC (d-f) and AIBL (g-i) data, respectively.

We found statistically significant differences in mean quality between 1.5, 3T* and 3T images on the ADNI dataset (Figure S5), as evaluated using SNR and BRISQUE. However, when NIQE was used, we found the difference 1.5 and 3T* images to be statistically significant but not between the 3T* and 3T images (Figure S5). We also found significant differences in mean quality using SNR, BRISQUE and NIQE between 1.5T and 3T* images on the NACC data (Figure S5). For the AIBL data, we found statistically significant differences in 1.5T and 3T* using SNR and NIQE but not using BRISQUE (Figure S5).


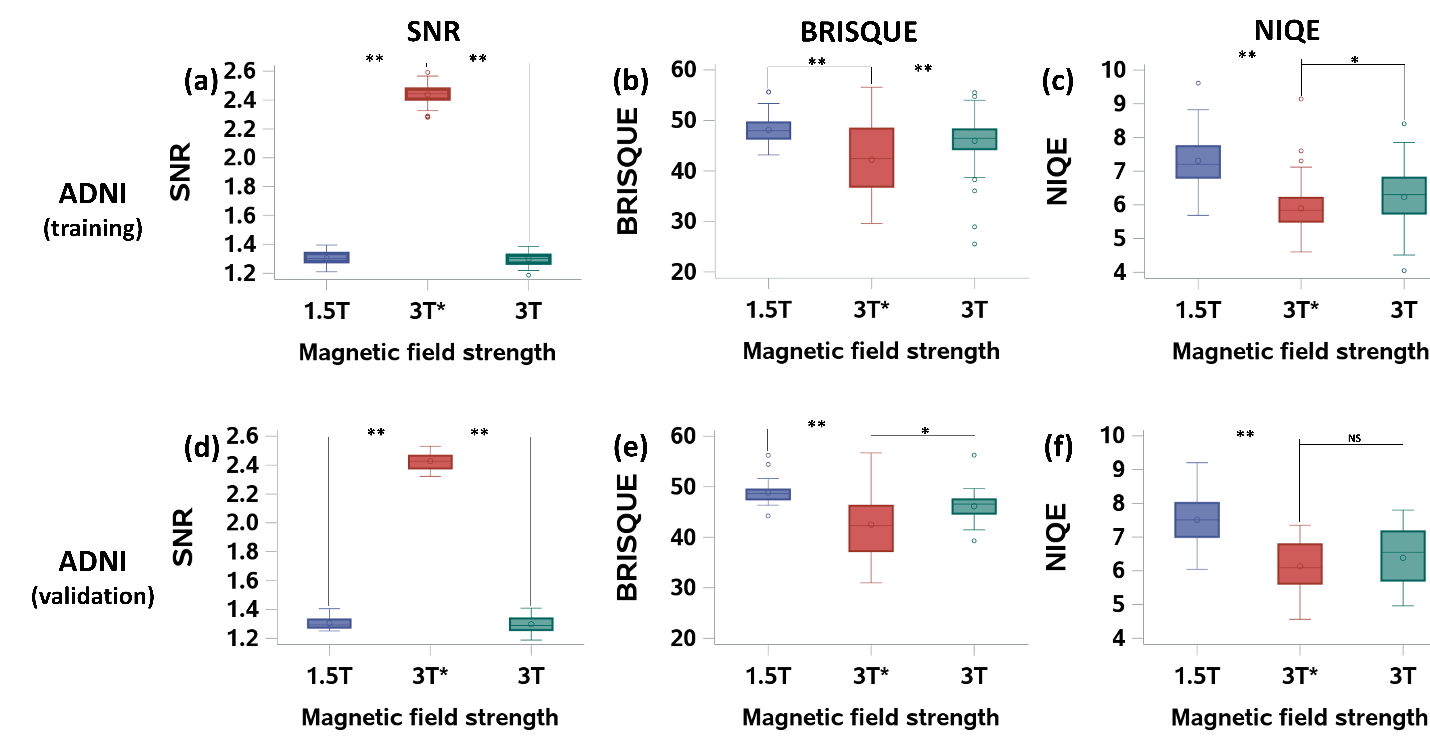


*: Statistical significance at p<0.05 level

**: Statistical significance at p<0.0001 level

Figure S6: ANCOVA analysis to assess the mean image quality using SNR, BRISQUE and NIQE for simpleGAN model in the ADNI-training (a-c) and ADNI-validation (d-f) data.

The effect of independent variables such as age, education, MMSE scores, and type of scanner was evaluated using a stepwise forward selection process and models were adjusted accordingly.

We found statistically significant evidence of an overall difference in mean image quality in the ADNI-training dataset using SNR, BRISQUE and NIQE measures between 1.5T, 3T* and 3T images (Figure S6). We also found statistically significant evidence of an overall difference in mean image quality in the ADNI-validation dataset using SNR and BRISQUE measures between 1.5T, 3T* and 3T images (Figure S6). However, a significant evidence of an overall difference in mean image quality using NIQE was not observed on the ADNI-validation data between 3T* and 3T images (Figure S6).

| **ADNI-training** | | | **ADNI-validation** | | **ADNI-testing** | |
| --- | --- | --- | --- | --- | --- | --- |
| **SNR** | | | | | | |
|  | **1.5T** | **3T** | **1.5T** | **3T** | **1.5T** | **3T** |
| **3T*** | <.0001 | <.0001 | <.0001 | <.0001 | <.0001 | <.0001 |
| **3T** | 0.89 |  | 0.81 |  | 0.93 |  |
| **BRISQUE** | | | | | | |
|  | **1.5T** | **3T** | **1.5T** | **3T** | **1.5T** | **3T** |
| **3T*** | <.0001 | 0.04 | <.0001 | 0.002 | <.0001 | 0.004 |
| **3T** | 0.005 |  | 0.03 |  | 0.07 |  |
| **NIQE** | | | | | | |
|  | **1.5T** | **3T** | **1.5T** | **3T** | **1.5T** | **3T** |
| **3T*** | <.0001 | 0.006 | <.0001 | 0.057 | <.0001 | 0.38 |
| **3T** | <.0001 |  | 0.006 |  | <.0001 |  |

Table S3: Specific group differences in magnetic field strength (1.5T, 3T, 3T*) were evaluated using Tukey's post hoc procedure across the image quality measures SNR, BRISQUE and NIQE for the simpleGAN model in the ADNI-training, testing and validation data.

Another series of analyses were performed to evaluate the mean difference in image quality generated by the simpleGAN model (Table S3). ANCOVA and Tukey's post hoc tests were performed on ADNI-training, ADNI-test and ADNI-validation data, t-test was performed on NACC and AIBL data (Table S3). The models were adjusted for age, education, MMSE scores, and type of scanner using a stepwise forward selection process (Table S3).
